# Combating the causative agent of amoebic keratitis, *Acanthamoeba castellanii*, using *Padina pavonica* alcoholic extract: toxicokinetic and molecular docking approaches

**DOI:** 10.1038/s41598-024-63691-8

**Published:** 2024-06-13

**Authors:** Sara S. Abdel-Hakeem, Faten A. M. Hassan, Awatief F. Hifney, Shimaa H. Salem

**Affiliations:** 1 Parasitology Laboratory, Zoology and Entomology Department, Faculty of Science, Assiut University, Assiut, 71526 Egypt; 2 Microbiology Department, Faculty of Science, Taiz University, Taiz, Yemen; 3 Botany and Microbiology Department, Faculty of Science, Assiut University, Assiut, 71526 Egypt

**Keywords:** *Acanthamoeba castellanii*, Amoebic keratitis, Cytochrome P450 monooxygenase, Molecular docking, ADME analysis, Parasitology, Microbiology, Zoology, Diseases, Eye diseases

## Abstract

Natural products play a significant role in providing the current demand as antiparasitic agents, which offer an attractive approach for the discovery of novel drugs. The present study aimed to evaluate in vitro the potential impact of seaweed *Padina pavonica* (*P*. *pavonica*) extract in combating *Acanthamoeba castellanii* (*A. castellanii*). The phytochemical constituents of the extract were characterized by Gas chromatography–mass spectrometry. Six concentrations of the algal extract were used to evaluate its antiprotozoal activity at various incubation periods. Our results showed that the extract has significant inhibition against trophozoites and cysts viability, with complete inhibition at the high concentrations. The IC_50_ of *P. pavonica* extract was 4.56 and 4.89 µg/mL for trophozoites and cysts, respectively, at 24 h. Morphological alterations of *A. castellanii* trophozoites/cysts treated with the extract were assessed using inverted and scanning electron microscopes and showed severe damage features upon treatment with the extract at different concentrations. Molecular Docking of extracted compounds against *Acanthamoeba* cytochrome P450 monooxygenase (AcCYP51) was performed using Autodock vina1.5.6. A pharmacokinetic study using SwissADME was also conducted to investigate the potentiality of the identified bioactive compounds from *Padina* extract to be orally active drug candidates. In conclusion, this study highlights the in vitro amoebicidal activity of *P. pavonica* extract against *A. castellanii* adults and cysts and suggests potential AcCYP51 inhibition.

## Introduction

*Acanthamoeba* spp. is an opportunistic protozoan parasite widely distributed and causes serious human infections, including amoebic keratitis (AK) and granulomatous encephalitis^1^.

It is prevalent in various aquatic sources such as brackish, marine, groundwater, drinking water, and swimming pool, as well as contaminated contact lens and cleaning solutions^2^. Globally, the prevalence of contact lens wear has raised the potential of eye infections, it is estimated that there are 1.5 to 2 million cases annually^3^. Approximately 85% of patients suffering from AK were contact lens users and the infection was related to poor hygiene^4^. Two strains of *Acanthamoeba* (*A. castellanii* and *A. polyphaga*) were isolated from soft contact lenses and disinfected solutions in Upper Egypt^5^. There are two stages of *Acanthamoeba* during the life cycle, the metabolically active trophozoite form and the cyst form. The cyst wall consists of the ecto- and endocyst, which are separated by a gap and primarily composed of D-glucopyranose and cellulose^6,7^. Ostiols are the areas where the two envelopes are in contact which have an operculum that could act as a gate for the excystment of trophozoite^8,9^. Due to the cyst walls and great resistance to unfavorable conditions, the cysts are more transmissible and have a longer lifespan. Thus, the inhibition of encystation during the treatment of amoebic infections can lead to more favorable outcomes. Although compounds such as chlorhexidine, polyhexamethylene, amphotericin B, ketoconazole, miconazole, clotrimazole, and fluconazole currently used to combat *Acanthamoeba* have been investigated empirically, they are surface-active^10^. The transformation of trophozoite to resistant cyst form is the main challenge during the treatment of *Acanthamoeba* infection^11^. Although there are available drugs that exhibited activity against *Acanthamoeba,* it is highly recommended to find alternative naturally occurring products to omit all potential safety concerns from synthetic chemicals^11,12^. Marine algae represent a novel source of bioactive compounds that proved various biological activities^13–17^. Seaweed contains several potent antioxidants such as carotenoids, phenols, terpenoids, and polysaccharides. Besides, it is a great source of protection against free radical damage^18^. The biological activities of *P. pavonica* (Brown seaweeds) as antivirals^19^, anti-inflammatories^20^, and antimicrobials^21^ have been studied. Therefore, we used in this study *P. pavonica* alcoholic extract as a novel, feasible, and economical anti-amoebic agent against Neff strain of *A. castellanii* (OL336245.1). In addition, we used molecular docking approach to evaluate the inhibition activity of bioactive compounds in the extract against *Acanthamoeba* cytochrome P450 monooxygenase as a vital enzyme for maintaining the membrane integrity. Recently several studies have used this approach to decipher the interaction mode of anti-amoebic agents and their target proteins. For example, eugenol derivatives were found to inhibit the active binding ability of *Acanthamoeba* that affects its mobility, shape, and viability^22^. Also, we evaluated the pharmacokinetic properties of these compounds using ADME analysis to highlight the safety and the potential of using this extract as an anti-amoebic agent. To the best of our knowledge, this research will be the first attempt to explore the mode of action of the extract as an alternative drug that is safe, inexpensive, and economically feasible.

## Materials and methods

### Cultivation of free-living *amoeba*

The Neff strain of *A. castellanii* (OL336245.1) was obtained from Parasitology Laboratory, Zoology and Entomology Department, Faculty of Science, Assiut University, Assiut, Egypt^5^. *Acanthamoeba* trophozoites were cultured in non-nutrient agar plates containing 0.12 g NaCl, 0.004 g MgSO_4_.7H_2_O, 0.004 g CaCl_2_·2H_2_O, 0.142 g Na_2_HPO_4_, 0.136 g KH_2_PO_4_, and 15.0 g agar/L of distilled water at pH 6.8 that seeded with a thin layer of live *Escherichia coli* (NNA-*E*. *coli*). The plates were incubated at 30 °C under standard atmospheric conditions and were observed daily for 7 days for trophozoites and 14 days for cysts^5,23,24^. For cysts formation, *A. castellanii* cells in their exponential growth were collected and washed three times in phosphate buffer saline solution (PBS) and incubated in Neff’s encystment medium (0.1 M KCl, 0.02 M Tris–HCl, 8 mM MgSO_4_, 0.4 mM CaCl_2_, 1 mM NaHCO_3_, pH 9.0) at 25 °C for 72 h. The cyst stages were harvested after two weeks and then washed three times in PBS by centrifugation at 3000 rpm for 10 min. Counting was done using a hemocytometer, and the number was adjusted to a final concentration of 1 × 10^4^ (> 95.0% trophozoites)/mL^25^.

### Identification of *P. pavonica* and preparation of the algal extract

The samples were collected from the inter-tidal region of the Red Sea shores, Egypt, between latitude 27–17–13″ N and longitude 33° 46′21″ E) in the summer of 2022. Seaweed was identified on the taxonomic database website created and maintained by Guiry^26^. Live and healthy seaweed samples were harvested manually and washed in seawater to remove all plankton on them from random plants, animal castings, sand, lime, and other adhering residue material. Then, the samples were washed with fresh water to remove salt. The biomass was dried in the shade to prevent thermal degradation or photolysis, and the dried materials were weighed, coarsely ground in a mechanical mill, and then stored in paper bags at 4 °C in the dark until used. 25 g of dried algal biomass was extracted with 70% ethanol by stirring for 24 h. The extract was then filtered and dried at 40 °C under reduced pressure using a rotary evaporator. The obtained powder was resuspended in sterile distilled water and preserved in the fridge until use.

### Gas chromatography-mass spectrometry (GC–MS) analysis of *P. pavonica* ethanolic extract

The phytochemical constituents of *P. pavonica* aqueous ethanolic extract were analyzed with GC–MS equipment at NAWAH Scientific, Almokattam, Cairo, Egypt, using gas chromatography–mass spectrometry instrument stands with the following specifications: Instrument: a TRACE GC Ultra Gas Chromatograph (THERMO Scientific Corp., USA), coupled with a thermal mass spectrometer detector (ISQ Single Quadrupole Mass Spectrometer). The GC/MS system was equipped with a TR-5 MS column (30 m × 0.32 mm i.d., 0.25 μm film thickness). Analyses were carried out using helium as a carrier gas at a flow rate of 1.0 mL/min and a split ratio of 1:10 using the following temperature program: 60 °C for 1 min; rising at 4.0 °C/min to 240 °C and held for 1 min. The injector and detector were held at 210 °C. A diluted sample (1:10 hexane, v/v) of 1 μL of the mixture was injected. Mass spectra were obtained by electron ionization (EI) at 70 eV, using a spectral range of m/z 40–450. The identification of compounds was achieved by library search on a Wiley 275 L GC/MS database (Thermo Fisher Technology, Waltham, Massachusetts, United States) and using AMDIS software (www.amdis.net), identified by its retention indices (relative to n-alkanes C8–C22) and mass spectrum matching to available authentic standards. Wiley spectral library collection and the National Institute of Standards and Technology (NIST) library database curves generated by running GC analysis of representative authentic compounds^24,27^.

### Amoebicidal activity of the extract against trophozoites and cysts

Six concentrations of *P. pavonica* ethanolic extract were prepared at 5, 25, 50, 75, 100, and 160 µg/mL in PBS and its amoebicidal effect was investigated by incubating 100 μL of the calibrated trophozoite/cyst suspension with 100 μL of serial dilution of the test concentrations in Eppendorf tubes and mixing well. 100 μL of the calibrated *A. castellanii* suspension was incubated with 100 µL (0.02%) chlorhexidine (CHX), as a reference drug (positive control). For untreated control, 100 μL of PBS was added to 100 μL of *A. castellanii* suspension. Each concentration was tested in triplicates and incubated at 26 °C at different periods (3, 6, 9, 12, and 24 h)^25^. The viability of *A. castellanii* trophozoite/cyst was assessed using 0.4% trypan blue, which selectively stains non-viable cells. The number of viable trophozoites or cysts was determined using a hemocytometer. The morphological changes of the trophozoites and cysts were observed using an inverted microscope (TE2000-S, Tokyo, Japan). The inhibitory percentage of *A. castellanii* trophozoite/cyst viability was calculated as follows:

Inhibitory percentage (%) = 100 × (mean number of viable trophozoites/cysts in untreated control − mean number of viable trophozoites/cysts in the treatment)/mean number of viable trophozoite/cyst in untreated control. IC_50_, the concentration to inhibit the growth of 50% of cells, was determined using nonlinear regression analysis. All experiments were conducted in triplicates and statistical analyses were performed to determine the cell viability percentage. Furthermore, the results of the cultures containing no viable cysts were confirmed by inoculating onto an NNA- *E. coli* plate at 30 °C for an additional three days and examining for any viable trophozoites or cysts^24^.

### Scanning electron microscopy (SEM)

The morphological alternations of *A. castellanii* trophozoite/cyst treated with different concentrations of *P. pavonica* extract were assessed compared to the control. Trophozoites and cysts treated with low concentrations of the extract (5, 25, 50 mg/mL) were examined after 24 h. The samples were collected by centrifugation at 3000 rpm for 5 min, re-suspended in PBS, and fixed with 2.5% glutaraldehyde overnight. Then, they were dehydrated with a series of graded alcohols (20%, 40%, 60%, 80%, 90%, and 100% ethanol), mounted on aluminum stubs, and allowed to dry using a critical point dryer. Samples were further coated with gold particles, and the morphology of *A. castellanii* trophozoites and cysts after the treatment was subsequently examined under a SEM Zeiss Leo 435 VP scanning electron microscope (Leo Electron Microscopy Ltd., Cooperation Zeiss Leica, Cambridge, England) at 15 kV with a magnification of 1500 to 2500 k for image capture in the Electron Microscope Unit at Assiut University, Egypt.

### Sequence analysis

Pairwise sequence alignment between *A. castellanii* strain Neff with a truncated transmembrane helix (AcCYP51) and human CYP51 was performed using the CLUSTAL Omega1.2.4 server (https://www.ebi.ac.uk/Tools/msa/clustalo/) to evaluate key amino acid conservancy between the two enzymes. The SIAS (Sequences Identities and Similarities) tool (http://imed.med.ucm.es/Tools/sias.html) was used to calculate the sequence identity between AcCYP51 and human CYP51. Amino acid sequences of AcCYP51 (ID: 6UX0) and human CYP51 (ID: 4UHL) were downloaded in FASTA format from the Protein Data Bank (https://www.rcsb.org/)^28^.

### Molecular docking

Molecular docking of extracted compounds and AcCYP51 was performed using Autodock Vina 1.5.6. The AcCYP51-Isavuconazole complex structure was retrieved from PDB with ID 6UX0^29^. Isavuconazole and water molecules were removed from the crystal structure, and polar hydrogens and Kollman charges were added. Isavuconazole-binding amino acids (Y114, F116, S117, F121, V126, T127, A290, F293, A294, L363, V366, M471, M365, and M367) were identified by analyzing the complex 3D structure. The binding energies of the tested compounds with the crystal structure of AcCYP51 were used to evaluate the binding affinities of the tested compounds. Top-scoring compounds were visualized using Chimera 1.12 software. Isavuconazole was redocked in the active site, and its binding energy was used as a reference for binding affinity evaluation.

### ADME and drug-likeness prediction

For evaluating the potentiality of compounds to be good drug candidates, the ADME analysis and drug-likeness parameters were performed using the free web tool SwissADME (Absorption, distribution, metabolism, and excretion) (http://www.swissadme.ch/)^30^. The chemical structure of the compounds was drawn using MarvinJs and illustrated in Fig. S3.

### Data analysis

The inhibitory percentage of *A. castellanii* trophozoite/cyst viability was calculated as follows:

Inhibitory percentage (%) = 100 × (mean number of viable trophozoites/cysts in untreated control − mean number of viable trophozoites/cysts in the treatment)/mean number of viable trophozoite/cyst in untreated control^31^.

IC_50_ was calculated through a non-linear sigmoidal curve fitting with the normalized response using GraphPad Prism 7 software. All experiments were repeated independently in triplicate. Data were expressed as mean ± standard deviation (SD). Statistical analysis of IC50 values Statistical differences were determined using the t-test with *P* ≤ 0.05 considered statistically significant.

### Ethical approval and consent to participate

The ethical approval was obtained from Faculty of Science Research Ethics Committee (FSREC), Assiut University, Egypt (proposal No. 1-2023-0007). The experimental protocols were in accordance with the ethical principles in the Parasitology Laboratory, Zoology and Entomology Department, Faculty of Science, Assiut University, Assiut, Egypt.

## Results

### GC–MS analysis

GC–MS analysis of *P. pavonica* ethanolic extract indicated the presence of active twenty-three phytochemical compounds as shown in Table 1. The phytochemical constituents of the extract cover a broad range of chemical structures, such as saturated and unsaturated fatty acids, esters, flavonoids, phenolics, terpenoids, lipids, and alkenes. Retention time (Rt), molecular formula (MF), molecular weight (MW), and concentration (peak area%) of the detected bioactive compounds are listed in Table 1. The biological activities of major compounds extracted from *P. pavonica* are summarized in Table S1.

**Table 1:** GC–MS spectral analysis of phytochemical compounds identified in an aqueous ethanol extract of *P. pavonica.*

**#**	Rt (min)	Compound name	Nature	MF	MW	Peak area %
1	22.17	1-Nonadecene	Alkene	C_19_H_38_	266	0.43
2	23.03	Tetradecanoic acid	Carboxylic acid	C_14_H_28_O_2_	228	3.36
3	23.56	9-Octadecenoic acid (z)-	Unsaturated fatty acid	C_18_H_34_O_2_	282	0.56
4	24.44	Neophytadiene	Terpenes	C_20_H_38_	278	4.01
5	24.54	1,3,5-Triazine-2,4-diamine,6-chloro-N-ethyl-	Heterocyclic	C_5_H_8_ClN_5_	173	1.25
6	24.94	3,7,11,15-Tetramethyl-2-hexadecen-1-ol	Terpene alcohol	C_20_H_40_O	296	1.35
7	25.97	Isochiapin B	Sesquiterpene	C_19_H_22_O_6_	346	0.39
8	26.23	Hexadecanoic acid, methyl ester	Fatty acid methyl ester	C_17_H_34_O_2_	270	1.41
9	27.06	n-Hexadecanoic acid	Fatty acid	C_16_H_32_O_2_	256	23.34
10	27.42	Tetraneurin A	Ester	C_17_H_22_O_6_	322.4	0.62
11	28.54	Palmitic Acid, TMS derivative	Fatty acid	C_19_H_40_O_2_Si	328	3.75
12	29.19	2-Acetyl-3-(2-cinnamido)ethyl-7-methoxyindole	Alkaloid	C_22_H_22_N_2_O_3_	362	0.44
13	29.51	2-Hydroxy-3-[(9E)-9-Octadecenoyloxy]Propyl (9E)-9-Octadecenoate #	Ester	C_39_H_72_O_5_	620	0.87
14	29.67	1-Dodecanol, 3,7,11-trimethyl-	Sesquiterpenoids	C_15_H_32_O	228	4.56
15	35.49	Flavone 5,7-OH,3',4'-OME	Flavonoid	C_17_H_14_O_6_	314	1.06
16	35.49	Strychane,1-acetyl-20à-hydroxy-16 methylene-	Alkaloid	C_21_H_26_N_2_O_2_	338	1.06
17	36.26	3',4',7-Trimethylquercetin	Flavonoid	C_18_H_16_O_7_	344	0.46
18	36.68	Bis(2-ethylhexyl) phthalate	Phthalic acid ester	C_24_H_38_O_4_	390	4.40
19	38.78	Flavone 4'-OH,5-OH,7-di-*O*-glucoside	Flavonoid glycoside	C_27_H_30_O_15_	594	0.65
20	39.83	Methyl (2S,12bR)-2-((Z)-1-hydroxybut-2-en-2-yl)-1,2,6,7,12,12b-hexahydroindolo[2,3-a]quinolizine-3-carboxylate	Heterocyclic	C_21_H_24_N_2_O_3_	352	1.22
21	41.17	Ethyl iso-allocholate	Steroidal derivatives	C_26_H_44_O_5_	436	0.86
22	43.29	6,8-Di-C-á-Glucosylluteolin	Fatty acid	C_27_H_30_O_16_	610	2.13
23	43.69	Cholest-5-en-3-ol (3á)-	Steroid	C_27_H_46_O	386	7.92

Rt; retention time, MF; molecular formula, MW; molecular weight.

### The amoebicidal and cysticidal effect of *P. pavonica* ethanolic extract

Six concentrations of *P. pavonica* were tested against *A. castellanii* trophozoites and cysts, separately after 3 h, 6 h, 9 h, 12 h, and 24 h. Statistically, the mean number of viable *A. castellanii* trophozoites and cysts was significantly reduced (P ≤ 0.0001) compared to the control (Tables 2 and 3), and complete inhibition was observed in the high concentrations. The inhibitory percentage of viable *A. castallanii* trophozoites was higher than cysts at the different incubation periods with the *P. pavonica* extract (Fig. S1). There was no significant decrease in the inhibitory percentage of the viable trophozoites (41.7%) at a low concentration of 5 µg/mL started from 9 h after the treatment (Fig. S1), compared with the inhibitory percentage in cysts (37.1%) (Fig. S2). After 12 h and 24 h, the viability reduction percentage of trophozoites and cysts treated with 25 µg/mL of the extract were 87.9% and 94.4% for trophozoites and 92.9% and 97.1% for cysts, respectively.

**Table 2:** Effects of different concentrations of *P. pavonica* ethanolic extract on the mean number of viable *A. castellanii* trophozoites compared to the control and reference drug at different incubation periods.

Treatments	Viable trophozoites at different incubation periods				
	After 3 h	After 6 h	After 9 h	After 12 h	After 24 h
Control	7.9 ± 2.1^aB^	7.5 ± 0.98^aB^	6.9 ± 0.9^aB^	7.3 ± 1.2^aB^	12.7 ± 0.6^aA^
5 µg/mL	4.77 ± 0.9^bB^	3.4 ± 0.8^bB^	4.0 ± 1^bB^	4.0 ± 1^bB^	8.26 ± 1^bA^
25 µg/mL	3.93 ± 0.7^bA^	2.1 ± 0.5^cB^	1.27 ± 0.3^cC^	0.89 ± 0.3^cC^	0.71 ± 0.08^cC^
50 µg/mL	3.2 ± 0.3^bA^	1.8 ± 0.4^cB^	0.73 ± 0.25^cdC^	0.39 ± 0.35^cCD^	0 ± 0^cD^
75 µg/mL	1.06 ± 0.2^cA^	0.27 ± 0.06^dB^	0.1 ± 0.17^dBC^	0 ± 0^cC^	0 ± 0^cC^
100 µg/mL	0.8 ± 0.17^cA^	0.07 ± 0.1^dB^	0 ± 0^dB^	0 ± 0^cB^	0 ± 0^cB^
160 µg/mL	0.33 ± 0.15^cA^	0 ± 0^dB^	0 ± 0^dB^	0 ± 0^cB^	0 ± 0^cB^
CHX	0 ± 0^c^	0 ± 0^d^	0 ± 0 ^d^	0 ± 0 ^c^	0 ± 0 ^c^
*P*	0.0000 ***	0.0000 ***	0.0000 ***	0.0000 ***	0.0000 ***
LSD	1.518	0.879	0.865	0.9735	0.762
Variance	0.769	0.258	0.25	0.316	0.194
R^2^	0.927	0.971	0.970	0.967	0.993
F	29.239	77.820	75.919	68.864	377.067

Data represents mean ± SD.

Small letters represented significantly between all conc. in all treatments, and Capital letters represented significantly one conc. effects between tested periods, according to Duncan's multiple-range test (*P* ≤ 0.05).

Values followed by the same letter are not statistically different according to Duncan’s multiple-range test (*P* ≤ 0.05).

CHX; chlorhexidine.

**Table 3:** Effects of different concentrations of *P. pavonica* ethanolic extract on the mean number of viable *A. castellanii* cysts compared to the control and reference drug at different incubation periods.

Treatments	Viable cysts at different incubation periods				
	After 3 h	After 6 h	After 9 h	After 12 h	After 24 h
Control	10.2 ± 1^aA^	8.8 ± 2.4^aA^	9.6 ± 3.4^aA^	7.3 ± 1.4^aAB^	4.9 ± 0.6^aB^
5 µg/mL	9.3 ± 1.6^aA^	6.2 ± 0.9^bB^	5.6 ± 0.6^bBC^	3.9 ± 0.9^bCD^	2.4 ± 1^bD^
25 µg/mL	5.6 ± 0.9^bA^	1.9 ± 0.6^cB^	1.6 ± 0.5^bcB^	0.49 ± 0.4^cC^	0.14 ± 0.25^cC^
50 µg/mL	4 ± 2.6^bcA^	1.4 ± 0.5^cdB^	0.9 ± 0.2^cB^	0.3 ± 0.4^cB^	0 ± 0^cB^
75 µg/mL	3.3 ± 1.5^bcA^	1.2 ± 0.7^cdB^	0.67 ± 0.2^cB^	0 ± 0^cB^	0 ± 0^cB^
100 µg/mL	2.07 ± 0.1^cdA^	0.47 ± 0.5^cdB^	0 ± 0^cC^	0 ± 0^cC^	0 ± 0^cC^
160 µg/mL	0.9 ± 0.7^dA^	0 ± 0^dB^	0 ± 0^cB^	0 ± 0^cB^	0 ± 0^cB^
CHX	0.07^dA^	0 ± 0^dB^	0 ± 0^cB^	0 ± 0^cB^	0 ± 0^cB^
*P*	0.0000 ***	0.0000 ***	0.0000 ***	0.0000 ***	0.0000 ***
LSD	2.331	1.707	2.150	1.070	0.734
Variance	1.813	0.973	1.543	0.382	0.179
R^2^	0.909	0.933	0.911	0.961	0.960

Data represents mean ± SD.

Small letters represented significantly between all conc in all treatments, and Capital letters represented significantly one conc. effects between tested periods, according to Duncan's multiple-range test (P ≤ 0.05).

Values followed by the same letter are not statistically different according to Duncan’s multiple-range test (P ≤ 0.05).

CHX; chlorhexidine.

**Figure 1:**
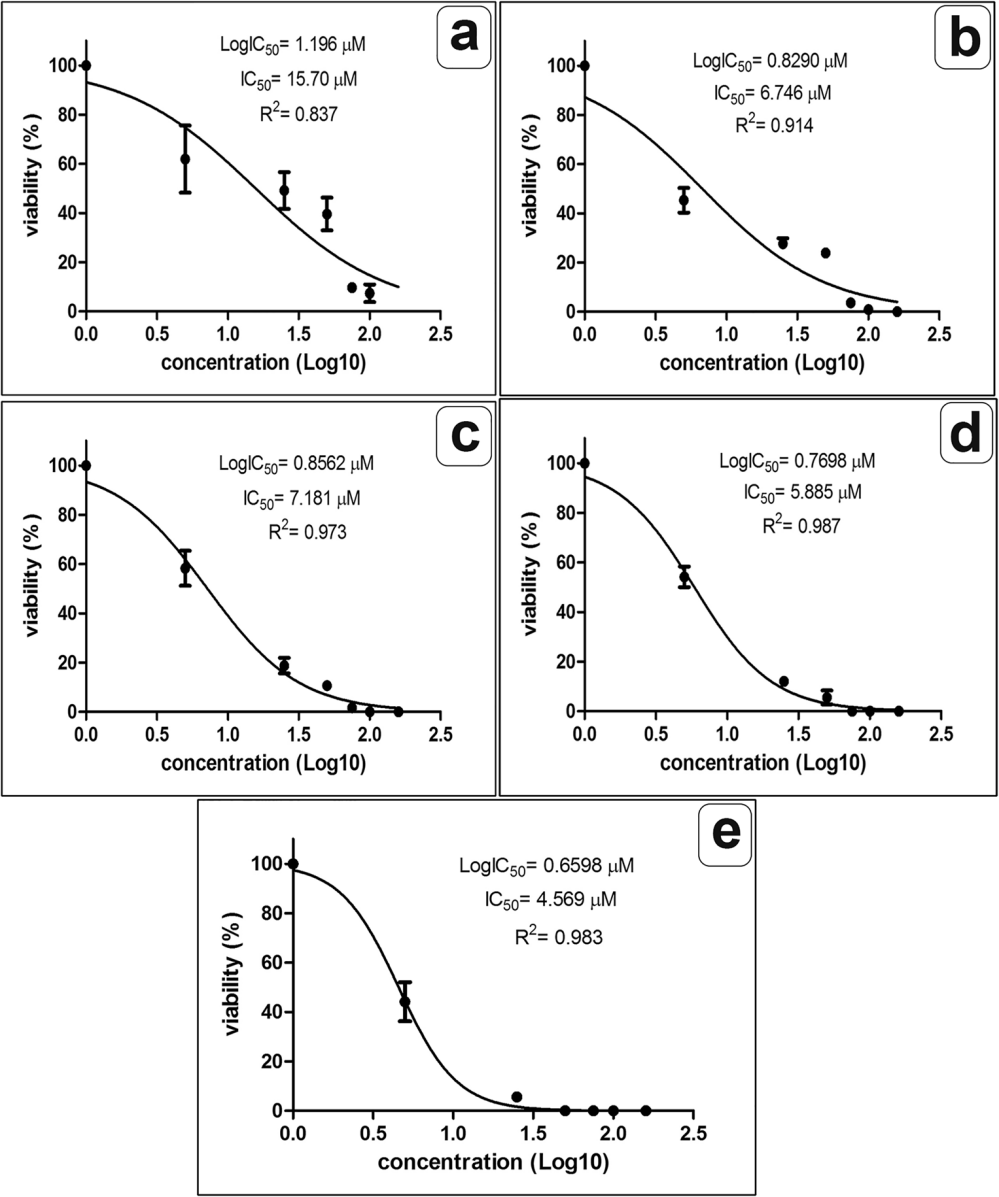
The lethal inhibition concentration (IC_50_) of *P. pavonica* ethanolic extract against *Acanthamoeba* trophozoites at different incubation periods 3 h, 6 h, 9 h, 12 h, and 24 h (**a**–**e**, respectively).

**Figure 2:**
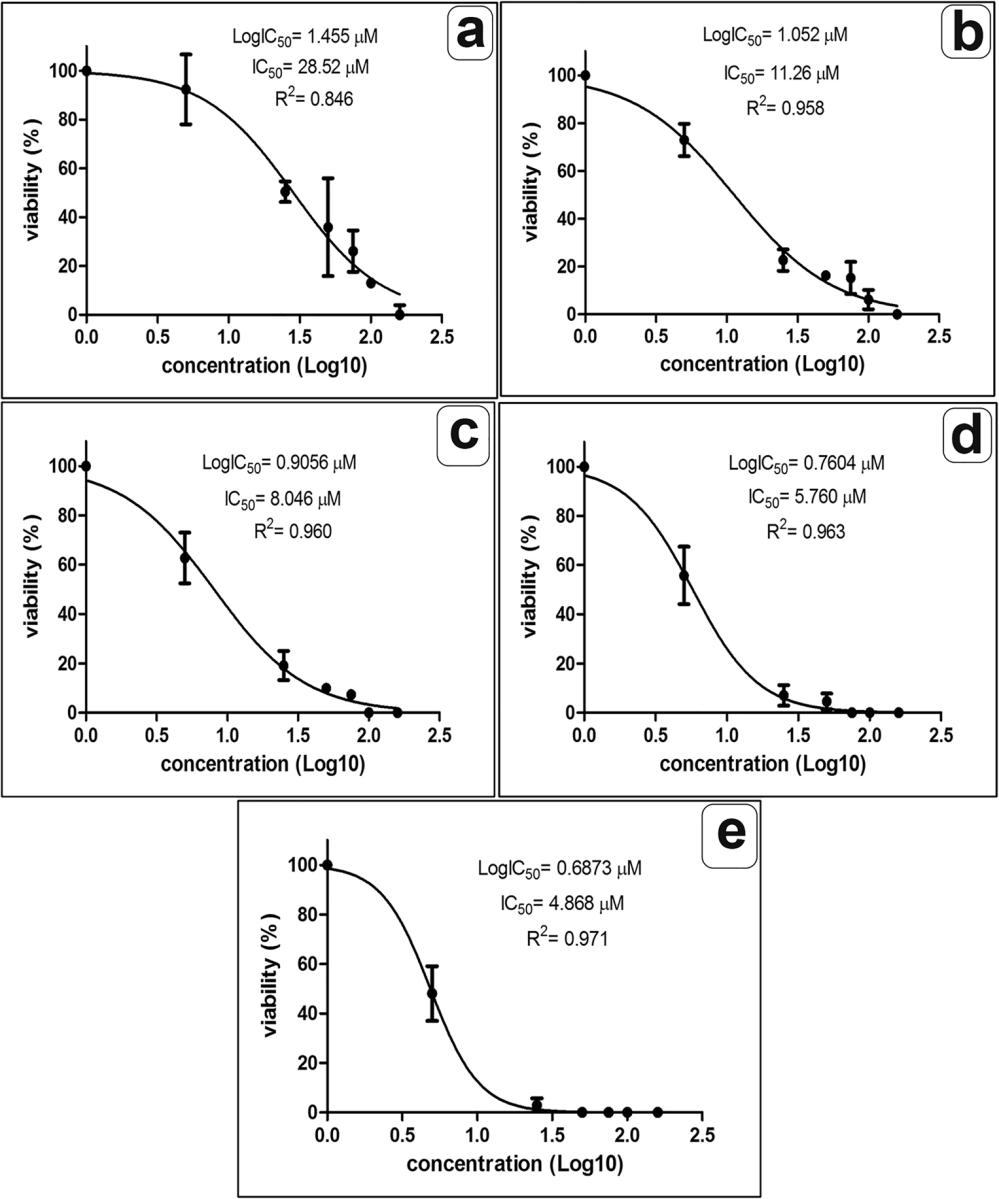
The lethal inhibition concentration (IC_50_) of *P. pavonica* ethanolic extract against *Acanthamoeba* cysts at different incubation periods 3 h, 6 h, 9 h, 12 h, and 24 h (**a**–**e**, respectively).

Figures 1 and 2 show the inhibitory concentration (IC_50_) of the extract against the trophozoites and cysts at different incubation periods. The IC_50_ values showed initial efficacy against the parasite stage after 3 and 6 h as follows: 15.70 µm and 6.7 µm for trophozoites; 28.5 µm and 11.26 µm for cysts. The most potent IC50 was observed after 24 h as follows 4.57 and 4.87 µm for trophozoite and cyst, respectively.

### Morphological alterations of *A. castellanii* trophozoites and cysts

**Figure 3:**
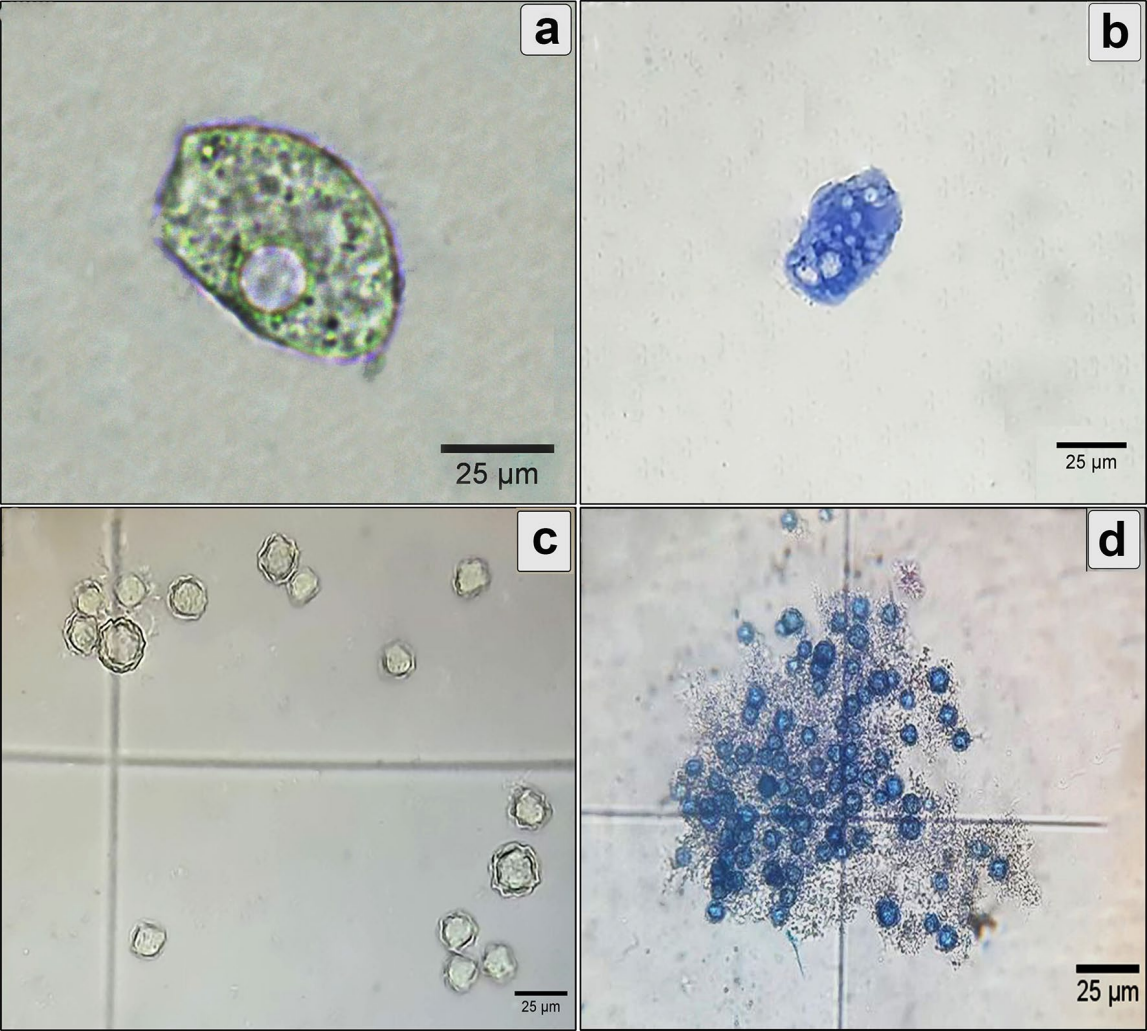
Effect of *P. pavonica* on the viability of *A. castellanii* trophozoites and cysts in comparison with untreated control. (**a**) Untreated trophozoite showed viable nucleus, definite contractile vacuole, and food vacuoles. (**b**) Treated trophozoite showed a shrinkage in the internal structures. (**c**) Untreated cysts with wrinkled ectocyst and smooth endocyst. (**d**) Treated cysts showed non-viable accumulated cysts with disintegration of surface architecture. (Trypan blue stain, × 400).

**Figure 4:**
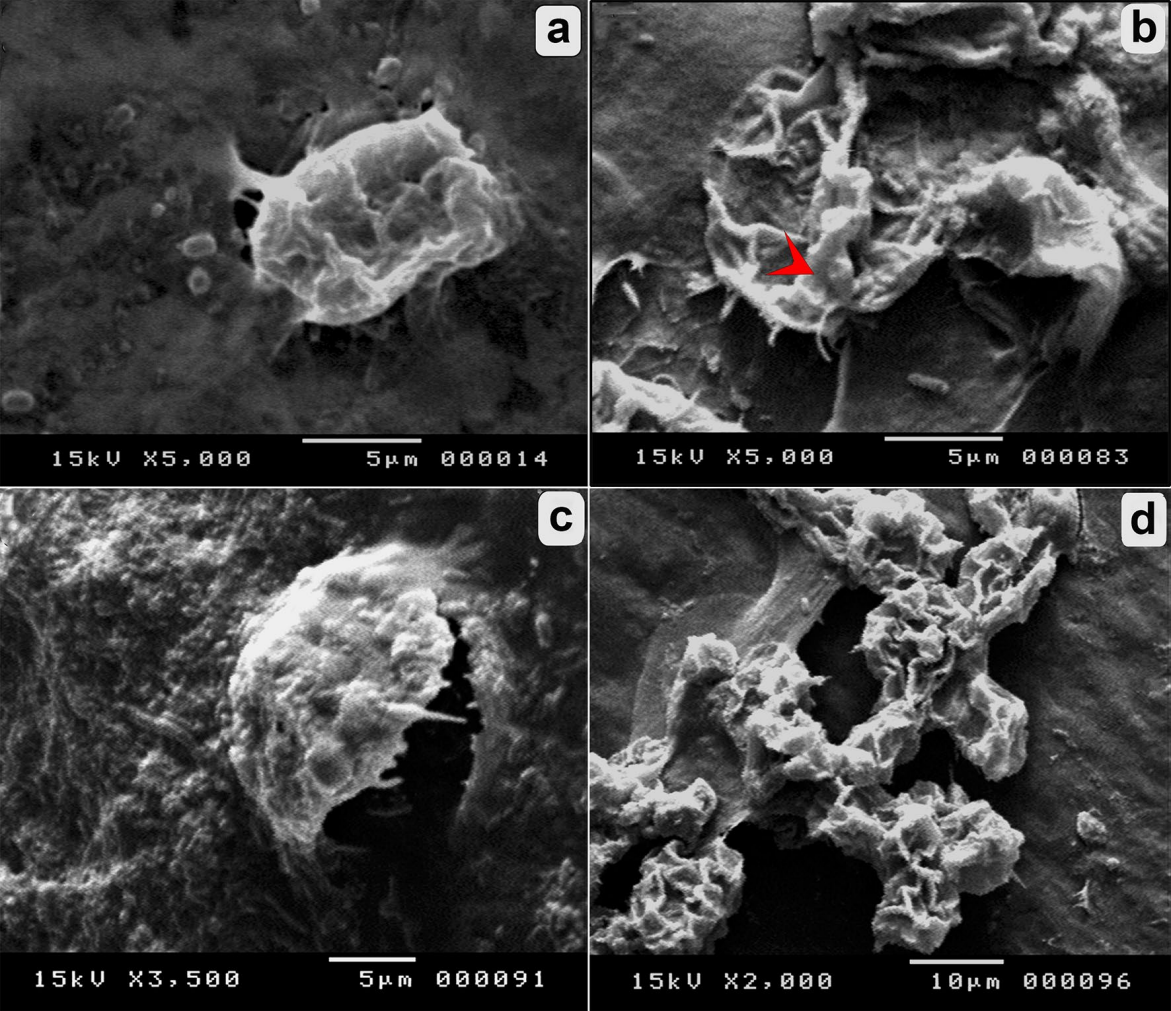
Scanning electron micrographs of *A. castellanii* trophozoites. (**a**) Untreated trophozoite with extensive acanthopodia. (**b**–**d**) Treated trophozoite showed degeneration (arrow) (**b**), lump shape and discretion from the surface (**c**) and collapsed trophozoites (**d**). Magnifications were revealed as: a, b = 5,000x; c = 3,500x; d = 2,000x.

**Figure 5:**
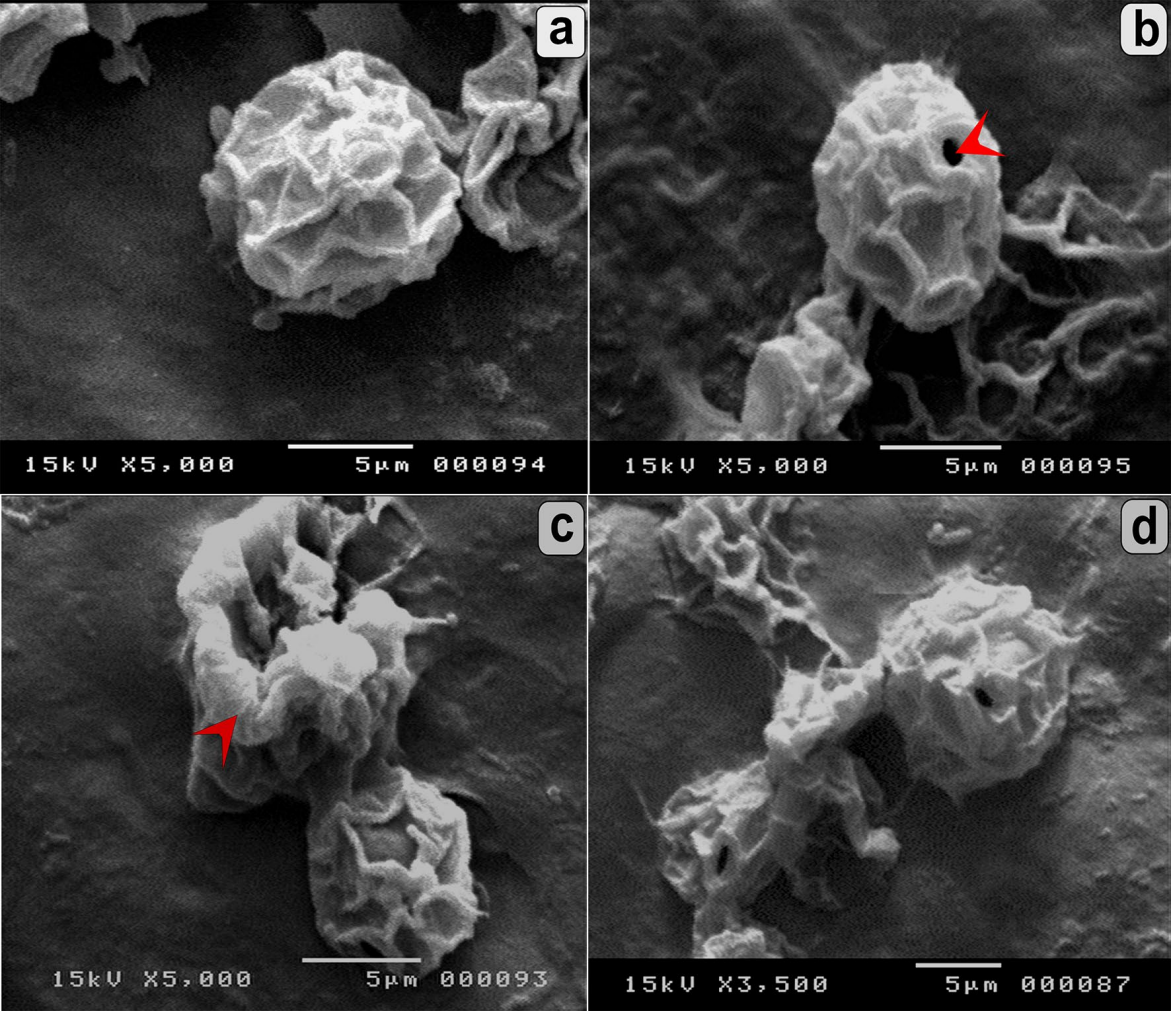
Scanning electron micrographs of *A. castellanii* cysts. (**a**) Untreated cyst was rounded with wrinkled ectocyst. (**b**,**d**) Treated cysts showed rupture in the operculum and pore formation (arrowhead) (**b**), distortion and progressive destruction of the cysts (arrow) (**c**,**d**). Magnifications were revealed as: a–c = 5,000x; d = 3500x.

Figure 3a shows untreated trophozoites having a unique shape characterized by acanthopodia and a large, bright contractile vacuole. The cysts were unicellular, rounded with a size range of 10–26 µm, with a wrinkled ectocyst and smooth endocysts (Fig. 3c). Overall, the activity of *P. pavonica* extract on *A. castellanii* viability was concentration- and time-dependent. In the treated groups, metabolically active trophozoites and cysts can pump trypan blue stain out and appear colorless (viable), whereas dead cells are stained blue with bright blue nuclei. Treatment with the different extract concentrations at different incubation times showed deformities in trophozoites and cyst shape and size. Reduction in trophozoite and cyst size, accumulation of deteriorated cells, shape deformation, and cell lysis were observed in different treated groups (Fig. 3b,d). Using SEM, untreated trophozoites were noted to be amoeboid in shape with many enveloping spikes that adhered to the surface (Fig. 4a). SEM showed deformity in the trophozoites treated with the extract in the form of shrinkage and degeneration (Fig. 4b). Interestingly, a clearly lump-like shaped cyst was observed (Fig. 4c), as well as the collapse of cells (Fig. 4d). The control cyst was rounded with a wrinkled ectocyst (Fig. 5a). Moreover, ruptured operculum and definite pore formation were sharply detected in the treated cysts, as shown in (Fig. 5b,c). Distortion and progressive destruction of *A. castellanii* cysts were reported (Fig. 5d).

### Sequence analysis and molecular docking

**Figure 6:**
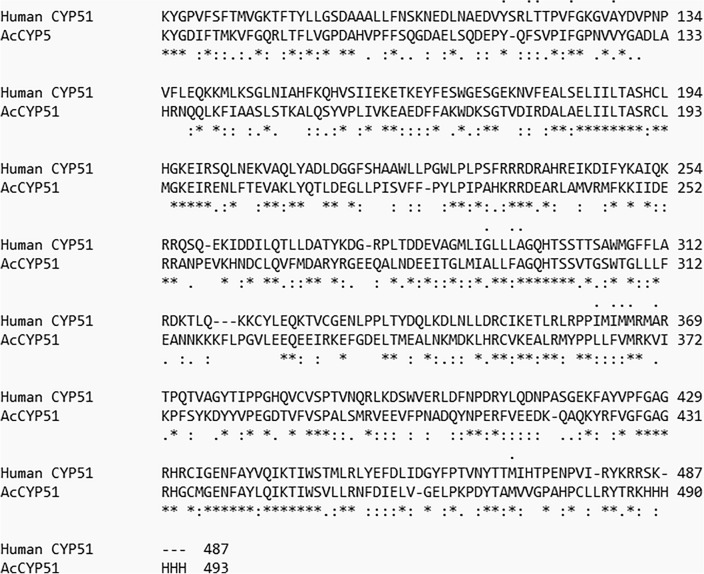
Pairwise sequence alignment between AcCYP51 and human CYP51. Symbols below sequences * identical, highly similar, semi-conserved amino acids. Symbols above sequences active residues.

**Figure 7:**
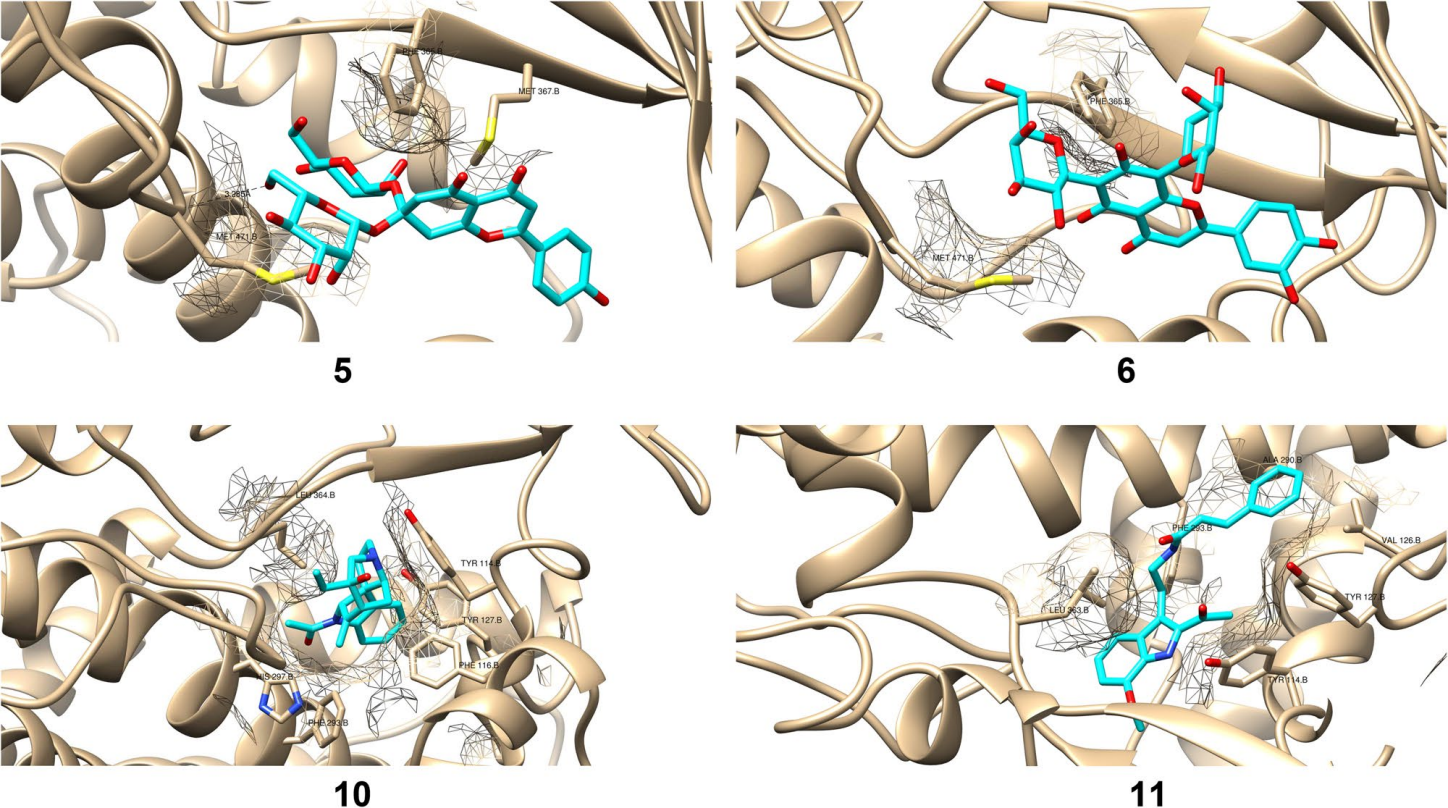
The 3D enzyme-drug complexes of the best-scored compounds (5,6,10,11). Gold sticks and mesh surfaces active amino acids; blue sticks compounds.

CYP enzymes belong to the heme-containing b-type monooxygenases superfamily. CYP51 (lanosterol 14α-demethylase) catalyzes an essential step in sterol biosynthesis, namely the conversion of 14α-methylsterols to ergosterols in fungi and cholesterol in humans. Ergosterol is the most abundant sterol in fungal cell membrane and regulates membrane permeability, fluidity, and integrity, therefore it has been a druggable target for antifungal drugs since the 1960s. Although *A. castellanii* strain Neff (AcCYP51) and human CYP51 have similar functions, pairwise sequence alignment revealed sequence homology of 6.72% and different active residues (Fig. 6). Among all key amino acids, only Y114, F121, A290, and M367 were conserved in both enzymes, therefore, AcCYP51-targeting drugs would have less inhibitory effect on human CYP51. Molecular docking of the tested compounds showed high binding affinities with AcCYP51 hydrophobic pocket amino acids in comparison to Isavuconazole (antifungal triazole). The Gibbs free energy of complexation was used to assess the stability of the enzyme-drug complex (Table 4). Figure 7 visualizes protein-drug interactions between the best-scored compounds and the active amino acids. Compound 5 forms a 3.2 Å-Hydrogen bond with Met471 through the primary hydroxyl group and undergoes hydrophobic interactions with Met471, Phe365, and Met367. Compound 6 makes hydrophobic interactions with Met471 and Phe365 at the active site. Compound 10 is fitted in the active site through hydrophobic bonds with Tyr114, Phe116, Tyr217 Phe293, HIS297, and Leu364. Compound 11 forms a stable complex with the hydrophobic pocket residues, Tyr114, Val126, Tyr127, Ala290, Leu363, and Phe293. Each compound reacts only with one conserved amino acid Y114 or M367, therefore, it is expected that the compounds do not inhibit human CYP51.

**Table 4:** Gibbs free binding energies of best-scored compounds with the AcCYP51 active site.

Number	Compound	Binding energy (KJ/mol)
1	1,3,5-triazine-2,4-diamine, 6-chloro-N-ethyl	− 5.22
2	Ethyl iso-allocholate	− 7.1
3	Flavone 5,7-OH,3',4'-OME	− 7.61
4	3',4',7-Trimethylquercetin	− 7.44
5	Flavone 4'-OH,5-OH,7- di-*O*-glucoside	− 7.88
6	6,8-Di-C-á-Glucosylluteolin	− 8.25
7	Methyl (2S,12bR)-2-((Z)-1-hydroxybut-2-en-2-yl)-1,2,6,7,12,12b-hexahydroindolo[2,3-a]quinolizine-3-carboxylate	− 7.3
8	Tetraneurin A	− 7.65
9	Isochiapin B	− 7.21
10	Strychanes	− 7.92
11	2-Acetyl-3-(2-cinnamido)ethyl-7-methoxyindole	− 8.21
12	Isavuconazole (Standard)	− 8.8

### ADME prediction analysis of bioactive compounds

**Figure 8:**
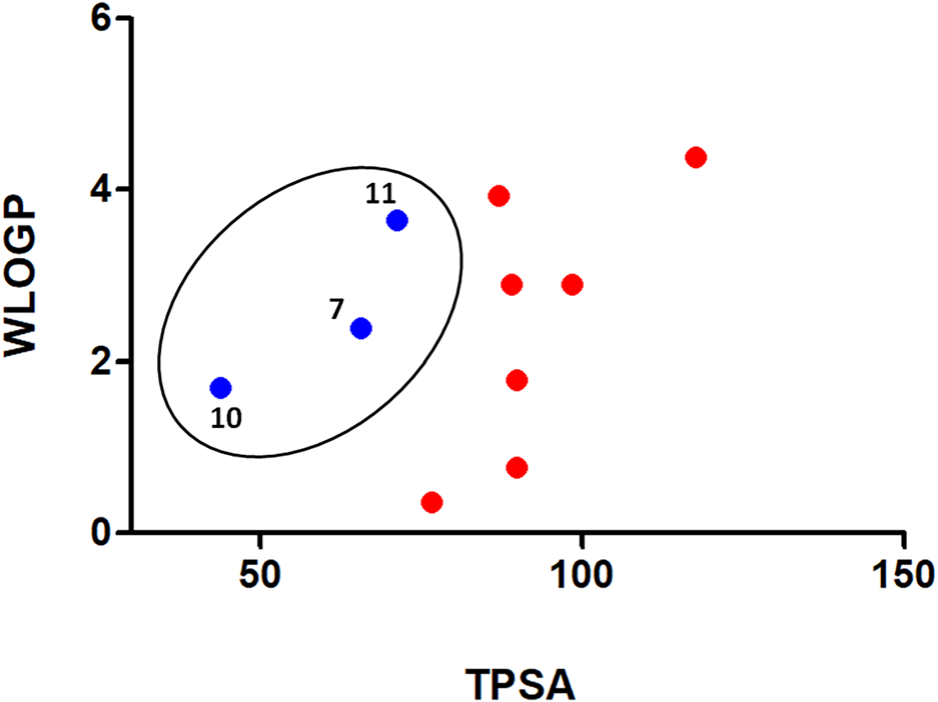
Boiled-Egg predictive model. Compounds with good evidence of brain access are represented by a blue color (7, 10, 11) while poorly absorbed molecules are represented by red color.

**Figure 9:**
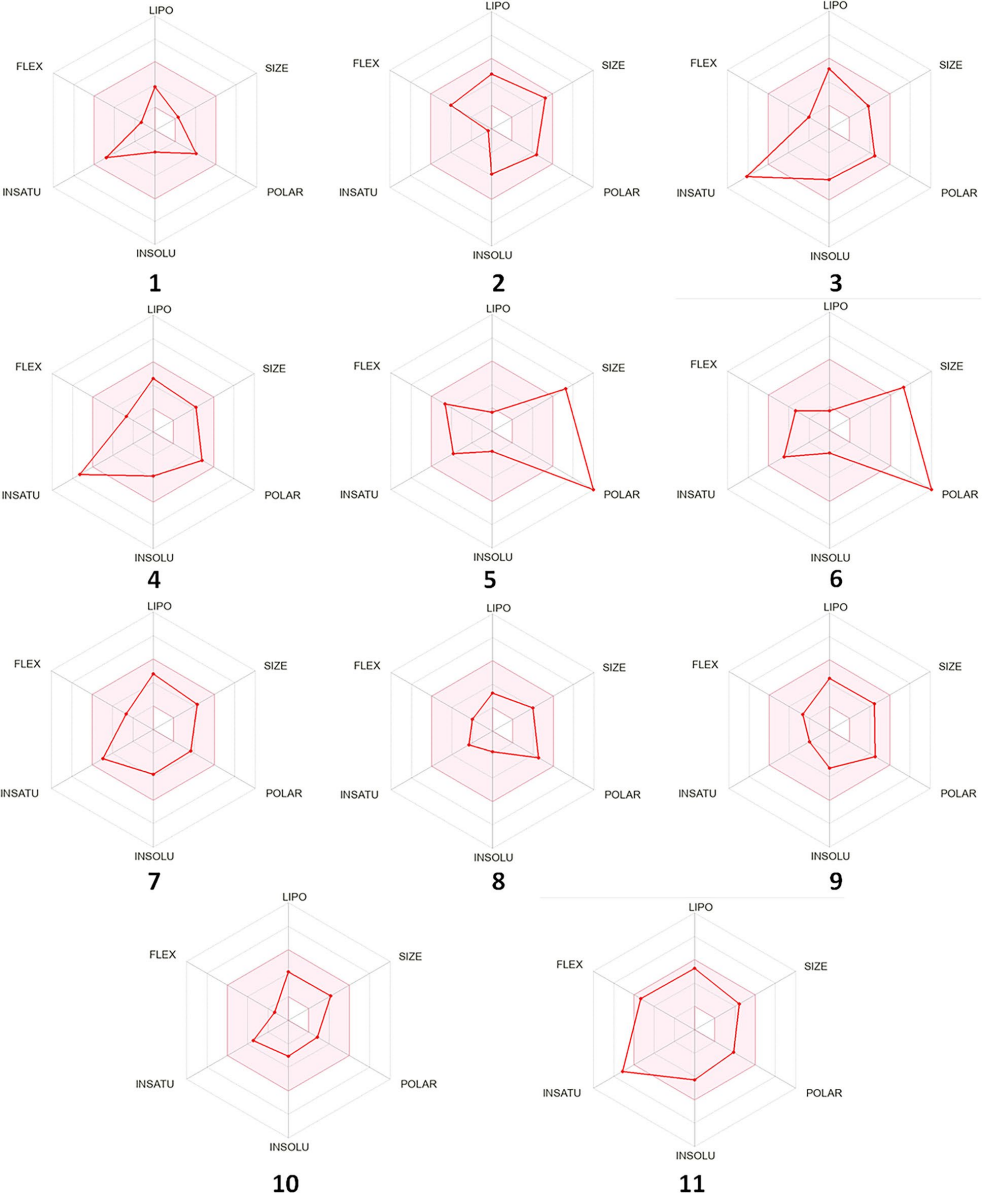
Bioavailability radar of bioactive drug-likeness compounds, where the pink areas are related to physicochemical properties (lipophilicity, molecular weight, solubility, and flexibility).

The pharmacokinetic properties of compounds that achieved good binding energy with the AcCYP51 active site were assessed using SwissADME online software and the results were summarized in Table 5. Nine of the examined compounds (1, 2, 3, 4, 7, 8, 9, 10, 11) do not violate Lipinski's rule and they have WLOGP values below 5.88 and TPSA below 131.6 Å^2^ so these compounds could be orally active drugs. According to the pharmacokinetic parameters, all compounds showed high gastrointestinal absorption except compounds 5 and 6. Besides, compounds 7, 10, and 11 can access the blood–brain barrier (BBB) as shown in Fig. 8. All compounds showed good bioavailability scores (0.55) except compounds 5 and 6 (0.17). The drug-likeness of the compounds can also be predicted via bioavailability radar (Fig. 9). As shown in Fig. 9, most of the compounds fall entirely in the pink area which represents the optimal range for each physicochemical property (lipophilicity, flexibility, saturation, solubility, polarity, and size), and these compounds have the potential to be drug-like, especially compounds 1, 2, 4, 7, 8, 9, 10, and 11.

**Table 5:** Predicted pharmacokinetics and ADME properties of the best-scoring compounds.

Properties	Compounds										
	1	2	3	4	5	6	7	8	9	10	11
Mol. Wt. (g/mol)	173.6	436.62	314.29	344.32	594	610.52	352.43	322.4	346	338.44	362.42
No. of rotatable bonds	2	6	3	4	7	5	4	3	4	2	8
No. of H-bond acceptors	3	5	6	7	16	16	3	6	6	3	3
No. of H-bond donors	2	3	2	2	10	12	2	1	1	1	2
TPSA (Å^2^)	76.72	86.99	89.13	98.36	269.43	291.43	65.56	89.9	89.9	43.78	71.19
Log Po/w (WLOGP)	0.36	3.93	2.89	2.9	− 3.2	− 3.34	2.38	0.77	1.79	1.7	3.64
Log Po/w (MLOGP)	− 0.27	3.46	0.47	0.17	− 4.71	− 4.97	2.05	0.95	1.66	2.72	1.91
GI absorption	High	High	High	High	Low	Low	High	High	High	High	High
BBB permeant	No	No	No	No	No	No	Yes	No	No	Yes	Yes
Lipinski’s violation	0	0	0	0	3	3	0	0	0	0	0
Bioavailability score	0.55	0.55	0.55	0.55	0.17	0.17	0.55	0.55	0.55	0.55	0.55

TPSA; molecular topological polar surface area, LogP; octanol–water partition coefficient, GI; gastrointestinal absorption, BBB; blood–brain barrier permeation.

## Discussion

Treatment against acanthamoebiasis requires several months due to the cyst stage. Medical intervention usually leads to an increase in adverse effects over time^32^. There have been many studies using medicinal herbs as alternatives against *Acanthamoeba* forms at low doses and with minimal toxicity^33,34^. Therefore, it was suggested that the combination of the active natural compounds with the current drugs could be an alternative protocol against *Acanthamoeba*. There are many commercial medications in use today; however, none of them are effective against all *Acanthamoeba* isolates. Besides, the cyst stage of *Acanthamoeba* spp. is resistant to environmental stressors such as starvation, temperatures, pH, osmolarity, irradiation, drugs^35^, and antimicrobial substances^36^. Because of their beneficial effects and high efficacy, seaweeds have acquired interest on a global scale in recent years. Our findings demonstrate the efficacy of a natural seaweed extract in combating *A. castellanii* stages. To overcome *A. castellanii*, the present study is focused on the anti-*Acanthamoeba* activity of *P. pavonica* ethanolic extract and the heterogenicity of the bioactive compounds of the extract with the chemical structure of the trophozoite and cysts using molecular docking and its pharmacokinetic. Our hypothesis explains the transformation of the trophozoite into a rigid double walls cyst during the treatment to protect itself from the effects of the medication. Based on the results, with increasing the extract concentration at a given time, the mean lethal percentage of trophozoites was higher than that of cysts. Furthermore, with an increased incubation period with the extract at each concentration, the lethal percentage, and the effectiveness of the extract in killing *Acanthamoeba* trophozoites and cysts were significantly increased, particularly at high concentrations of 160 μg/mL. The recorded effect of *P. pavonica* extract may be attributed to its alkaloids, flavonoids, fatty acids, and tannins, which are responsible for its biological activity^37^. Bioactive compounds in seaweed have strong antimicrobial, antiviral, antioxidant, antitumor, and anticancer properties^16,38,39^.

Different methods have been used for assessing the viability of *Acanthamoeba* cells following treatment with test compounds, with variations in IC_50_ between assays. The mean IC_50_ values were reported in the present study. Damiani et al.^40^ demonstrated the highest sensitivity for cytotoxicity detection, delivering the lowest IC_50_ values for all the substances. Our results revealed that *A. castellanii* trophozoites and cysts were completely dead at the 24 h incubation period (IC_50_/24 h was 4.6 µM and 4.9 µM, respectively). Lorenzo-Morales et al.^41^ reported that some *Laurencia oxasqualenoid* metabolites, such as iubol and dehydrothyrsiferol, had potent activities against *A. castellanii* Neff, with IC_50_ values of 5.30 and 12.83 µM, respectively. Further, the hydroxylated congeners thyrsiferol and 22-hydroxydehydrothyrsiferol were active against *A. castellanii* Neff at IC_50_ of 13.97 and 17.00 µM^41^. Due to varying mitochondrial functions, lysosomal activity, and membrane integrity, a reliable and uniform toxicity profile was not achieved^40^. Because of these variations, we used SEM to report the impact of the extract on the morphology of *A. castellanii* trophozoites and cysts. Skryabina et al.^42^ reported that the insufficiency of medical therapy frequently occurs due to the scanty absorption of local anti-*Acanthamoebic* drugs through thickened walls. Our findings showed rupture in the operculum and definite pore formation in the cysts treated with the extract, which might facilitate the diffusion and absorption of the extract. Data in the present study showed a positive impact of the extract, as mentioned above, on *A. castellanii*, which established that the extract is rich in many biologically active components. Most of these compounds from previous studies have antioxidants, antimicrobial, antifungal, anti-inflammatory, and anticancer properties^15,43,44^, nematicides, hepatoprotective^45,46^, and insecticides^17^. We performed molecular docking of the identified compounds with AcCYP51 active sites amino acids. Our results revealed that the tested compounds showed high binding affinities with AcCYP51 hydrophobic pocket amino acids in comparison to Isavuconazole (antifungal triazole). Isavuconazole inactivates AcCYP51 by a unique mechanism different from conventional enzyme active site inhibition. Isavuconazole binding to AcCYP51 causes N-termini swapping, formation of a nonplanar protein–protein interface, disordered 74 N-termini residues, irreversible loss of structural integrity, and proteolytic degradation. Isavuconazole binds to heme iron through a coordination bond and the active site residues through hydrophobic interactions. Although the tested compounds underwent no coordination bonds with heme iron, they formed stable complexes with the enzyme hydrophobic active site. In addition, similar binding modes with the active amino acids suggest potential inhibition of AcCYP51 by the extracted natural products.

Lipinski’s rule is one of the essential bioinformatics tools for rational drug design and determining drug-likeness. This rule was used to evaluate the pharmacokinetic properties of the identified bioactive compounds in *P. pavonica* ethanolic extract. According to Lipinski’s "Rule of Five", compounds should satisfy three out of the following five criteria in order to have drug potential: 1) molecular weight not more than 500 Da; 2) maximum of 10 hydrogen bond acceptors; 3) maximum of 5 hydrogen bond donors; 4) the number of rotatable bonds not less than 10; 5) log P value (lipophilicity) not greater than 4.15. WLOGP should be less than 5.88 and TPSA should be less than 131.6 Å^2^ for an orally available medication^47^. Using the free online software tool SwissADME, the pharmacokinetic properties of the compounds that achieved the best docking results were predicted (Table 5). Nine of the examined compounds (1, 2, 3, 4, 7, 8, 9, 10, 11) are thought to be potentially orally active drugs since they adhere to Lipinski’s criteria and have WLOGP values below 5.88 and TPSA below 131.6 Å^2^, respectively.

## Conclusion

Our results showed that nine compounds mainly have a potential effect on combating *A. castellanii* in vitro, which represents a promising alternative amoebicidal drug. The present results provide cornerstone information on the medicinal properties of the marine brown algae *P. pavonica* and its viability as an oral supplement drug. Further studies about the antiparasitic activities of marine brown alga and evaluation of its systematic mode of action are recommended. In addition, more chromatographic studies are required in the future to isolate and purify these bioactive compounds separately and assess their biological activity.

### Supplementary Information

Supplementary Information.

## Data Availability

All data analyzed or generated for the research are included in the article.
