# Supplementary material for: Combating the causative agent of amoebic keratitis, Acanthamoeba castellanii, using Padina pavonica alcoholic extract: toxicokinetic and molecular docking approaches
Source: Sci Rep. 2024 Jun 13;14:13610. doi: 10.1038/s41598-024-63691-8 (PMC11176165; doi:10.1038/s41598-024-63691-8)
Supplement: Supplementary file 1 — Supplementary Information. [file 41598_2024_63691_MOESM1_ESM.docx]

**Combating the causative agent of amoebic keratitis, *Acanthamoeba castellanii*, using *Padina* *pavonica* alcoholic extract: toxicokinetic and molecular docking approaches**

**Authors and affiliations:**

**Sara S. Abdel-Hakeem^1^, Faten A.M. Hassan^2^, Awatief F. Hifney^3^_,_ Shimaa H. Salem^3*^**

^1^ Parasitology Laboratory, Zoology and Entomology Department, Faculty of Science, Assiut University, 71526, Assiut, Egypt. ([sara_assiut86@aun.edu.eg](mailto:sara_assiut86@aun.edu.eg), **ORCID: 0000-0003-1069-5806**)

^2^ Microbiology Department, Faculty of Science, Taiz University, Taiz, Yemen

^3^ Botany and Microbiology Department, Faculty of Science, Assiut University, 71526, Assiut, Egypt

^*^ Corresponding author at Department of Botany and Microbiology, Faculty of Science, Assiut University, Assiut, Egypt 71526. Tel: +201067657884

E-mail: [shimaa.hassan@aun.edu.eg](mailto:shimaa.hassan@aun.edu.eg) (Shimaa H. Salem, PhD).

**ORCID ID: 0000-0002-2965-8143**

**Table S1.** Biological activities of some compounds identified in the ethanolic extract of *P. pavonica*

| **Compounds** | **Biological activity** | **References** |
| --- | --- | --- |
| 1-Nonadecene | Antifungal and anticancer activities | [1] |
| Tetradecanoic acid | Antimicrobial activities, antifungal, antioxidant, cancer preventive, nematicide, and hypercholesterolemic | [2] [3] |
| 9-Octadecenoic acid (z)- | Antibacterial activity | [2] |
| Neophytadiene | Antimicrobial activity | [4] |
| 3,7,11,15-Tetramethyl-2-hexadecen-1-ol | Antioxidant, antimicrobial, and anti-inflammatory. | [5] [6] |
| Isochiapin B | Stabilize free radicals. | [7] |
| Hexadecanoic acid, methyl ester | Antioxidant activity. | [8] |
| n-Hexadecanoic acid | Antioxidant, pesticide, flavor, 5-Alpha reductase-inhibitor, antifibrinolytic, hemolytic, lubricant, nematicide and anti-alopecia, anti-inflammatory, antispasmodic, anticancer and antiviral | [9] |
| Tetraneurin A | Antifeedant and pesticide | [10] |
| Palmitic Acid, TMS derivative | Hepatoprotective and anticancer activity. | [11] |
| 2-Acetyl-3-(2-cinnamido)ethyl-7-methoxyindole | Anticancer, antioxidant, antiviral and antimicrobial activities. | [12] |
| 2-Hydroxy-3-[(9E)-9-Octadecenoyloxy] Propyl (9E)-  9-Octadecenoate # | Antimicrobial ‏ activities. | [13] |
| 1-Dodecanol, 3,7,11-trimethyl- | Antimicrobial ‏ activities. | [13] |
| Flavone 5,7-OH,3',4'-OME | Antioxidant and anticancer activity ‏ | [14] |
| 3',4',7-Trimethylquercetin | Enhance the vasorelaxant activity. | [15] |
| Bis(2-ethylhexyl) phthalate | Antimicrobial and cytotoxic activity. | [16] |
| Ethyl iso-allocholate | Antimicrobial, diuretic, anti-inflammatory, and antiasthma. | [17] |
| 6,8-Di-C-á-Glucosylluteolin | Antioxidant, antiviral, antifungal, antibacterial and anticancer. | [18] |
| Cholest-5-en-3-ol (3á)- | Antibacterial activity. | [2] |
| 3',4',7-Trimethylquercetin | Antioxidant and antimicrobial activities; cancer enzyme inhibitors in pharmaceutics | [19] |


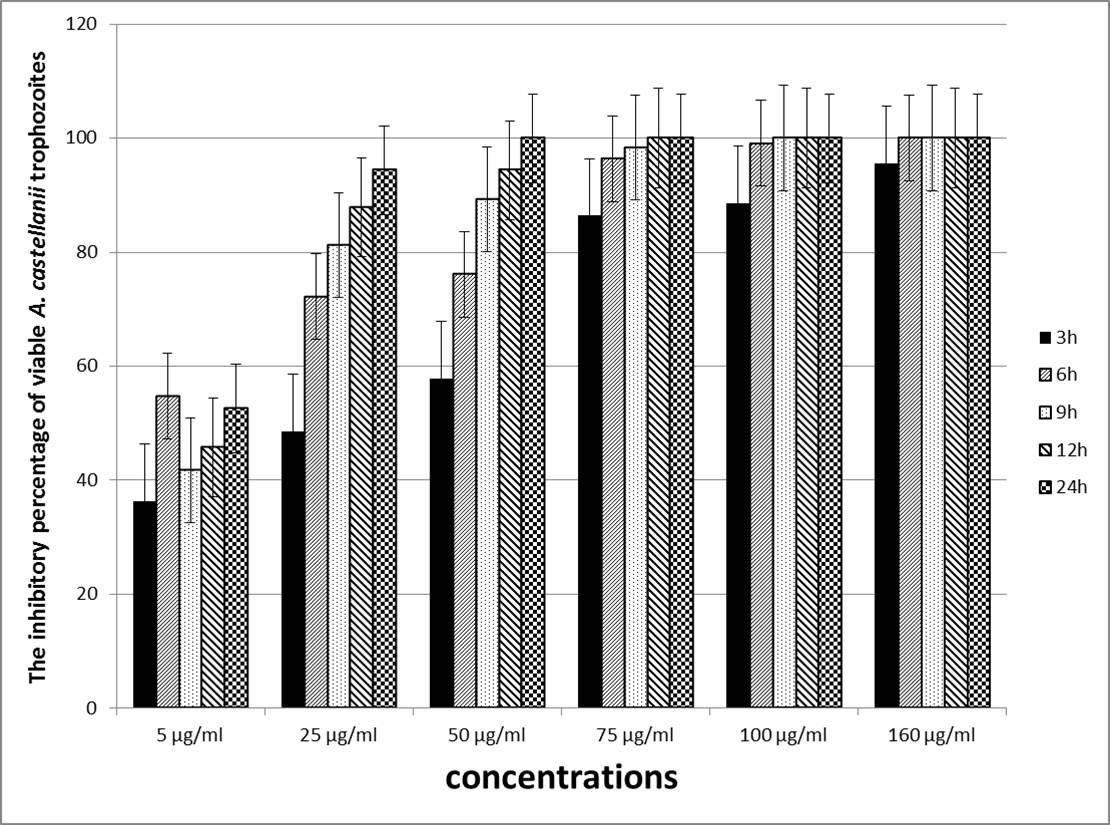


**Fig. S1.** The inhibitory percentages of viable *Acanthamoeba* trophozoites
incubated with different concentrations of *P. pavonica* ethanolic extract compared to
untreated control at different incubation periods.


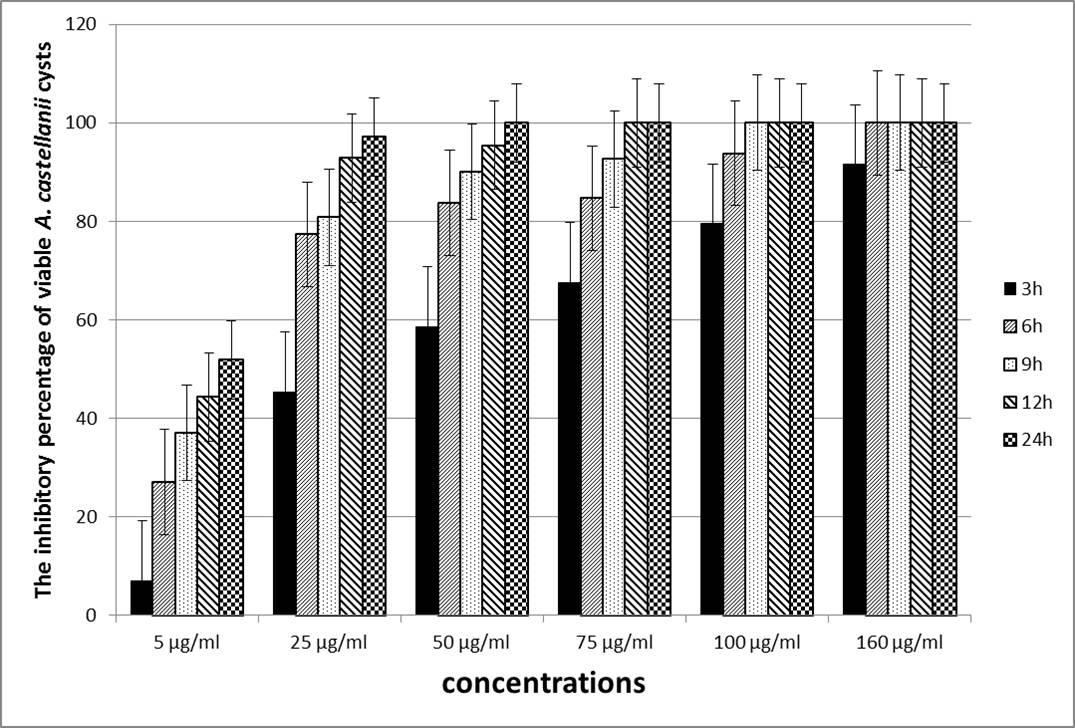


**Fig. S2.** The inhibitory percentages of viable *Acanthamoeba* cysts
incubated with different concentrations of *P. pavonica* ethanolic extract compared to
untreated control at different incubation periods.


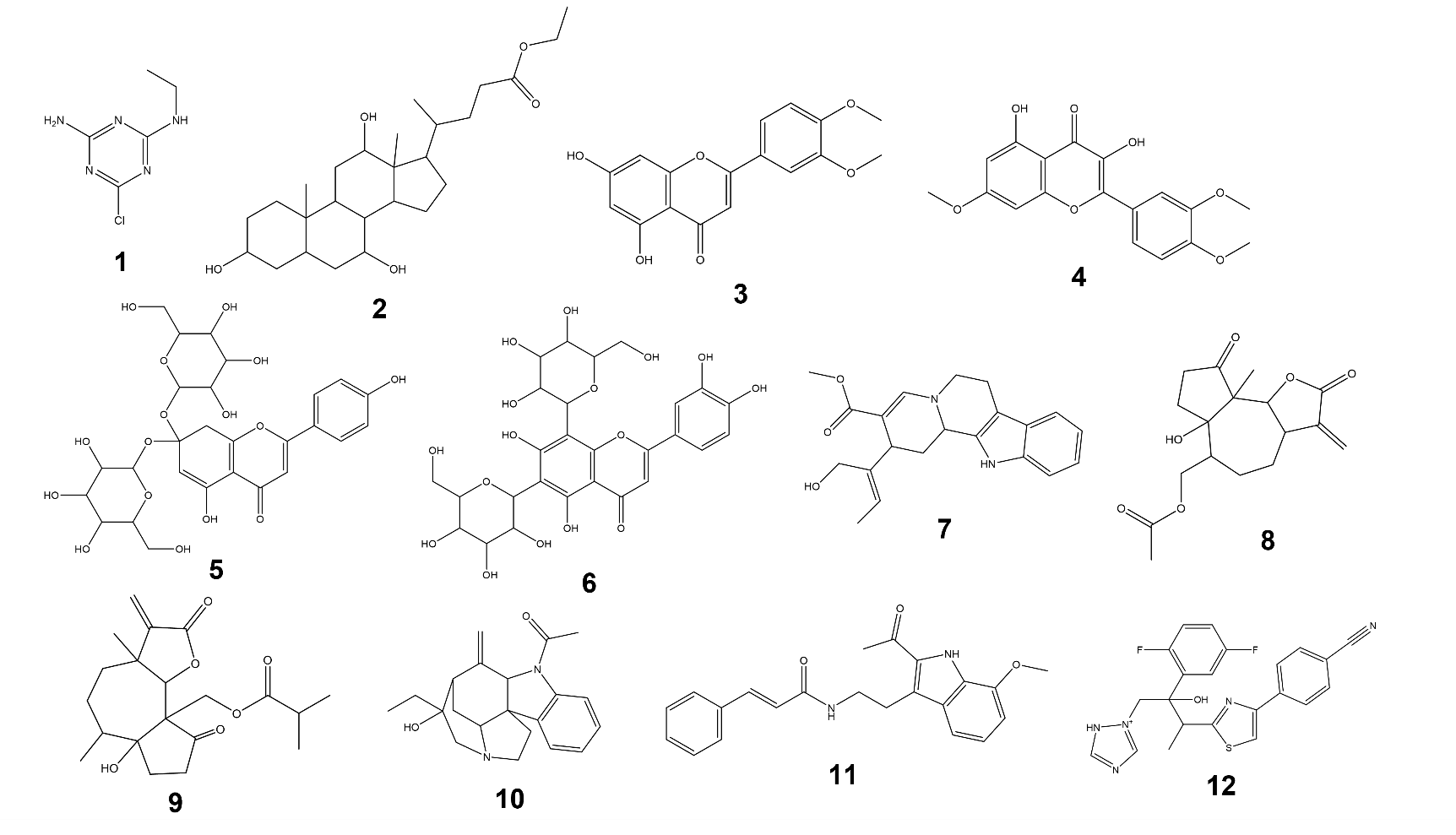


**Fig. S3.** Chemical structures of best-scored compounds with the AcCYP51 active site

[1] Sahin, N., Kula, I., Erdogan, Y., Investigation of antimicrobial activities of nonanoic acid derivatives. *Fresenius Environmental Bulletin* 2006, *15*, 141-143.

[2] Mujeeb, F., Bajpai, P., Pathak, N., Phytochemical evaluation, antimicrobial activity, and determination of bioactive components from leaves of Aegle marmelos. *BioMed research international* 2014, *2014*.

[3] Kim JungEun, K. J., Seo JiHye, S. J., Bae MinSuk, B. M., Bae ChunSik, B. C.*, et al.*, Antimicrobial constituents from Allium hookeri root. 2016.

[4] Ceyhan-Güvensen, N., Keskin, D., Chemical content and antimicrobial properties of three different extracts of Mentha pulegium leaves from Mugla Region, Turkey. 2016.

[5] Vijisaral Elezabeth, D., Arumugam, S., Analysis of bioactive constituents from organic crude ethanol extracts from the local medicinal plant of Cassytha filiformis L (Lauraceae) by gas chromatography-mass spectrometry. *Int J Pharm Sci Rev Res* 2014, *28*, 220-223.

[6] Susanto, N. S., Potency of Local Gracilaria sp. *Extract as an Antibacterial against Skin Disease Pathogen. Sch Acad J Biosci* 2021, *8*, 215-222.

[7] Espinoza, R. V., Peñarreta, J., Quijano-Avilés, M., Lucas, A. B.*, et al.*, Antioxidant activity and GC-MS profile of Conyza bonariensis L. leaves extract and fractions. *Revista Facultad Nacional de Agronomía Medellín* 2020, *73*, 9305-9313.

[8] Sudha, T., Chidambarampillai, S., Mohan, V., GC-MS analysis of bioactive components of aerial parts of Kirganelia reticulata Poir (Euphorbiaceae). *Journal of Current Chemical and Pharmaceutical Sciences* 2013, *3*, 113-122.

[9] Al, M. J., Mohammed, G. J., Anti-bacterial, Antifungal Activity and Chemical Analysis of Punica grantanum (Pomegranate peel) Using GC-MS and FTIR Spectroscopy.

[10] Abdelhamid, M. S., Kondratenko, E. I., Lomteva, N. A., GC-MS analysis of phytocomponents in the ethanolic extract of Nelumbo nucifera seeds from Russia. *Journal of applied pharmaceutical science* 2015, *5*, 115-118.

[11] Pal, L. C., Agrawal, S., Gautam, A., Chauhan, J. K., Rao, C. V., Hepatoprotective and antioxidant potential of phenolics-enriched fraction of Anogeissus acuminata leaf against alcohol-induced hepatotoxicity in rats. *Medical Sciences* 2022, *10*, 17.

[12] Qanash, H., Yahya, R., Bakri, M. M., Bazaid, A. S.*, et al.*, Anticancer, antioxidant, antiviral and antimicrobial activities of Kei Apple (Dovyalis caffra) fruit. *Scientific Reports* 2022, *12*, 5914.

[13] Shahin, A., Nabil-Adam, A., Elnagar, K., Osman, H., Shreadah, M. A., Bioactivity and metabolomics fingerprinting characterization of different organic solvents extracts of Padina pavonica collected from Abu Qir Bay, Egypt. *Egyptian Journal of Chemistry* 2022, *65*, 207-225.

[14] El-Fayoumy, E. A., Shanab, S. M., Gaballa, H. S., Tantawy, M. A., Shalaby, E. A., Evaluation of antioxidant and anticancer activity of crude extract and different fractions of Chlorella vulgaris axenic culture grown under various concentrations of copper ions. *BMC Complementary Medicine and Therapies* 2021, *21*, 1-16.

[15] Guerrero, M., Puebla, P., Carrón, R., Martin, M., Román, L. S., Quercetin 3, 7-dimethyl ether: a vasorelaxant flavonoid isolated from Croton schiedeanus Schlecht. *Journal of pharmacy and pharmacology* 2002, *54*, 1373-1378.

[16] Habib, M. R., Karim, M. R., Antimicrobial and cytotoxic activity of di-(2-ethylhexyl) phthalate and anhydrosophoradiol-3-acetate isolated from Calotropis gigantea (Linn.) flower. *Mycobiology* 2009, *37*, 31-36.

[17] Muthulakshmi, A., Mohan, V., GC-MS analysis of bioactive components of Feronia elephantum Correa (Rutaceae). *Journal of Applied Pharmaceutical Science* 2012, 69-74.

[18] Alqurashi, A. S., Al Masoudi, L. M., Hamdi, H., Abu Zaid, A., Chemical Composition and Antioxidant, Antiviral, Antifungal, Antibacterial and Anticancer Potentials of Opuntia ficus-indica Seed Oil. *Molecules* 2022, *27*, 5453.

[19] SAYIK, A., YUSUFOĞLU, A. S., Leyla, A., Türker, G.*, et al.*, DNA-binding, biological activities, and chemical composition of wild growing Epilobium angustifolium L. extracts from Canakkale, Turkey. *Journal of the Turkish Chemical Society Section A: Chemistry* 2017, *4*, 811-840.
